# Changes in immunization coverage and contributing factors among children aged 12–23 months from 2000 to 2019, Ethiopia: Multivariate decomposition analysis

**DOI:** 10.1371/journal.pone.0291499

**Published:** 2023-09-13

**Authors:** Melash Belachew Asresie, Gizachew Worku Dagnew, Yibeltal Alemu Bekele

**Affiliations:** Department of Reproductive Health and Population Studies, School of Public Health, College of Medicine and Health Sciences, Bahir Dar University, Bahir Dar, Ethiopia; Jimma University, ETHIOPIA

## Abstract

**Background:**

Immunization has been promoted as a global strategy aimed at improving child survival. The World Health Organization strives to make immunization services available to everyone, everywhere to save over 50 million lives by 2030. Monitoring the change and identifying the factors contributing to the change in immunization coverage over time and across the nations is imperative for continuing global success in increasing immunization coverage. In this study, we examined the changes and factors that contributed to the change in full immunization coverage over time in Ethiopia (2000 to 2019).

**Methods:**

We analyzed data on children aged 12–23 months, extracted from the 2000 and 2019 Ethiopian Demographic and Health Survey (EDHS) datasets. A total of 3,072 weighted samples (2,076 in 2000 and 966 in 2019) were included in the analysis. A multivariate decomposition analysis technique was used to determine change and identify factors that contributed to the change over time. Statistical significance was defined at a 95% confidence interval with a p-value of less than 0.05.

**Results:**

There was a 29.56% (95% CI: 24.84, 34.28) change in full immunization coverage between the two surveys. It increased from 14.62% (95% CI: 12.43, 17.11) in 2000 to 44.18% (95% CI: 37.19, 51.41) in 2019. The decomposition analysis showed that about 75% of explained change was attributed to the differences in the composition of explanatory variables (the endowment effect). Particularly, women aged 35–49 years (-2.11%), those who attended four or more antenatal care visits (17.06%), individual who had postnatal care visits (16.90%), households with two or more under-five children (2.50%), and those with a history of child mortality (17.80%) were significantly attributed to the change. The rest, 25% of the explained change was attributed to the difference in the effects of explanatory variables (coefficient). The change in the coefficient for women who had experienced child death (-20.40%) was statistically significant to the change in full immunization coverage over time.

**Conclusion:**

The finding of this study revealed that there was a statistically significant change in full immunization coverage over time. The majority of the change was attributed to the differences in the composition of explanatory variables such as antenatal care and postnatal care visits, age of the mother, and number of living children in the household. Therefore, strengthening maternal health services utilization may enhance immunization coverage in Ethiopia. Furthermore, the difference in coefficient of mothers with a history of child death had a substantial counteracting effect on the change, emphasizing the importance of raising awareness and delivering vaccine education to them and the larger community.

## Introduction

Child health improvements have been the center of global public health efforts over the last four decades [1]. Child mortality reduction has been one of the cornerstones of the global health agenda through Millennium Development Goals (MDGs) and Sustainable Development Goals (SDGs) [2]. Immunization is a key cost-effective public health intervention, towards attaining SDG3, the reduction of under-five mortality to less than 25 per 1000 live births by 2030 [3]. It averts an estimated 2 to 3 million deaths each year from vaccine-preventable illnesses across the world [4, 5]. Immunization not only saves infant lives, but it also boosts productivity, educational attainment, and economic growth [6, 7]. The World Health Organization (WHO) launched the Expanded Programme on Immunization (EPI) in 1974 as a public health initiative aimed at improving child health [5]. The immunization coverage rate has been successfully increasing from 5% at the inception to 86% in 2019 [5]. Despite these efforts, an estimated 18.2 million infants didn’t receive the initial dose of the Diphtheria-Pertussis-Tetanus (DPT) vaccines in 2021, as well as 6.8 million children were vaccinated partially in the same year [8].

The problem of low immunization coverage is deeply rooted in low and middle-income countries, more than 60% of these children unvaccinated or under-vaccinated in 2021 live in 10 low and middle-income countries (Angola, Brazil, the Democratic Republic of the Congo, Ethiopia, India, Indonesia, Myanmar, Nigeria, Pakistan, and the Philippines) [8]. Africa has the lowest immunization coverage, with 76% of children receiving third DPT doses, compared to 86% globally [8]. A systematic review study conducted in Sub-Saharan Africa showed that immunization coverage varies across the countries, ranging from 13 to 88% [9].

Ethiopia launched the EPI program in 1980 and offers it free at the community and facilities levels [10]. About 78% of children aged 12–23 months received the third dose of DPT in 2019, which was 18% in 2000 [11, 12]. Currently, one dose of Bacillus Calmette-Guerin (BCG), three doses of Oral Polio Vaccine (OPV), three pentavalent doses (Diphtheria-Pertussis-Tetanus, Hepatitis B, and Haemophilus Influenza type B vaccine), three doses of the Pneumococcal Conjugate, two doses of Rota-Virus, and one dose of Measle vaccines are recommended for children in their first year of life [10].

The Ethiopian federal Ministry of Health hoped to achieve 90% full immunization coverage at the national level and 80% in every district with all recommended vaccines among children aged 12–23 months by 2020 [13]. Besides, EPI has been one of the 18 components of the Health Extension Program, which has been offered at the community level by community health workers since 2005 [14]. However, only 43% of children aged 12–23 months received the recommended basic vaccines in 2019 (one dose of BCG, three doses of DPT and polio, and one dose of measles) [11]. As a result, Ethiopia is still one of the countries with the highest child mortality rates in the world [11] and vaccine-preventable diseases still account for the majority of causes of death [15].

Even though it remains below the national target, immunization coverage in Ethiopia has increased over the previous two decades, it has raised by 67% in the last two decades, from 14% in 2000 to 43% in 2019 [11, 12]. However, the driving factors for the observed change over time have not been thoroughly investigated. Indicate that identifying the sources and factors contributing to the change in full immunization over time is crucial to decision-makers and other important stakeholders participating in immunization programs, as it enables them to assess the past and ongoing efforts and develop suitable strategies to improve childhood immunization coverage towards achieving the goals of the WHO available immunization services to everyone, everywhere by 2030, to save 50 million lives [16] and SDG3, reducing under-five mortality to less than 25 deaths per 1000 live births in 2030 [3]. Decomposition analysis based on nationally representative data is paramount to examine the change and identify the driving factors for change [17]. Therefore, this study aims to identify the determinant of change in full immunization coverage among children aged 12–23 months over time (2000 and 2019) using the decomposition analysis technique.

## Methods

### Data source

We used the 2000 and 2019 EDHS data. At the time of the survey, administratively, Ethiopia was divided into nine regions and two administrative cities [11, 12].

### Study design and sampling technique

The EDHS was a national, regional, and residential-representative cross-sectional community-based survey. The EDHS used a two-stage stratified cluster sampling technique. The Ethiopia Population and Housing Census (PHC), which was conducted in 2007, served as the sampling frame for the EDHS. The census frame was a comprehensive list of the 84,915 enumeration areas (EAs) established for the 2007 PHC. It includes information about the EA’s location, types of residence, and the expected number of residential households in each region. Each region was initially stratified into urban and rural areas, yielding 21 sampling strata. Enumeration areas (EAs) were chosen in the first stage with a probability proportional to EA size based on Ethiopia’s 2007 PHC. In the second stage, a fixed number of 28 households per cluster were selected using an equal probability systematic selection method. The datasets we used for this objective were the "children’s record data (kids file)," from EDHS 2000 and 2019, which could be accessed on the DHS program website. All women between the ages of 15 and 49 who were either permanent residents of the selected households or visitors who slept in the household the night before the survey were asked about their children’s immunization status (aged 12–23 months) [11, 12].

### Study population and sample size

The study participants for this study were children aged 12 to 23 months. A total of 3,169 weighted children between the ages of 12 and 23 months were identified in the two surveys (2,143 in 2000 and 1026 in 2019). However, 97 children were excluded from the analysis due to missing observations in certain crucial variables. Finally, 3,072 weighted children were included in the analysis (2076 in 2000 and 966 in 2019) [11, 12].

### Measurements

The outcome variable of this study was children’s immunization status was classified dichotomously as "Yes" or "No." A child who received one dose of Bacillus Calmette-Guerin (BCG), three doses of combined Diphtheria-Pertussis-Tetanus (DPT), three doses of polio (excluding polio zero), and one dose of measle was considered as fully immunized or coded as "Yes (1)" and not fully immunized or coded as "No (0)" if a child had no received or missed any of the basic vaccines mentioned above [11, 18]. Initially, each vaccine antigen variable was classified as "1" or “Yes” for a child who received the vaccine dose and "0" or “No” for those who didn’t receive it. These values were then added together to calculate the level of immunization status. If a child received all of the recommended vaccine doses (one dose of BCG, three doses of DPT, three doses of polio, and one dose of measle), the immunization status was re-coded as "1" (fully immunized) and "0" (not fully immunized) if at least one of the vaccine doses was missing or not received any vaccines. The immunization status of children was obtained from written vaccination records including immunization cards or the mother’s verbal report if cards were not available.

#### Grouping variable

The year of the survey (2000 or 2019) was used to categorize study participants.

#### Explanatory variables

Based on their availability in two EDHS data sets and the literature, the following variables were chosen as exploratory variables [19]. Mothers’ age at the time of the interview (15–24, 25–34, and 35–49 years), region (developed, developing, and city administrative), place of residence (rural and urban), mother’s educational status (no education, primary, and secondary or above), religion (Christian, and other (Muslim, tradition, and others)), marital status (single and married), number of under-five children in the household (≤1 and ≥2), sex of children (female and male), birth order of index baby (1, 2–3, and 4 or above), place of delivery during index baby (home and health facility), ANC visits during index baby (no, 1–3 visits, and 4 or more visits), PNC visit during index baby (no and yes) and history of child death (yes and no).

Region: Amhara, Oromia, Tigray, and South Nation and Nationality People regions were grouped as developed, Benishangul Gumz, Harari, Gambella, and Afar region were grouped as developing and Addis Ababa and Dire Dewa city were grouped as city administrative.

### Data analysis

Using the "append" command in STATA software version 15.1, the 2019 EDHS data was appended to the 2000 EDHS data. To adjust disproportionate sampling and non-response, normalized weight was used. We also used the "svyset" command to handle the effect of the complex sample survey in EDHS. We recorded the variable "region" to be similar before merging the two datasets because it was not identical in the two datasets. After joining the two datasets, variables were recoded to create new categories.

Multivariate decomposition analysis was done using the “mvdcmp” command to understand the change and identify factors contributed for the change in full immunization coverage between two surveys (2000 and 2019). Multivariate decomposition analysis is important technique to detect the change and identify the source and the factor contributed to change the between the two surveys [17].

For this analysis, EDHS 2000 was coded as “0” and EDHS 2019 as “1”. Logit-based decomposition analysis technique was used to understand the change in the change in full immunization coverage and to identify the sources and factors that contributed to the change over the two surveys. The difference in the composition of explanatory variables (endowment) and the effect of the explanatory variables (coefficient) between 2000 and 2019 was considered to understand the sources of factors contributing to the change over time. Hence, the observed change in full immunization coverage between 2000 and 2019 EDHS was decomposed into characteristics (E) and Coefficients (C). The logit-based difference was decomposed as

$$Y=F(\frac{e^{x\beta }}{1+e^{x\beta }})$$ (1)

$$Ya‐Yb=F(\frac{e^{xa\beta a}}{1+e^{xa\beta a}})‐F(\frac{e^{xb\beta b}}{1+e^{xb\beta b}})$$ (2)

$$Y=[F(\frac{e^{xa\beta a}}{1+e^{xa\beta a}})‐F(\frac{e^{xb\beta a}}{1+e^{xb\beta a}})]+[F(\frac{e^{xb\beta a}}{1+e^{xb\beta a}})‐F(\frac{e^{xb\beta b}}{1+e^{xb\beta b}}]$$ (3)

where: Y is the dependent variable, X is the independent variable, β is the coefficient, and F is a differential logistic function of X and Y.

Before multivariate decomposition analysis, multi-collinearity between each independent variable was checked. Statistical significance was determined at a p-value of less than 0.05.

### Ethical considerations

The International Review Board of Demographic and Health Surveys (DHS) program data archivists waived informed consent and authorized us to download the data set for this study after the consent paper was submitted to the DHS program. The data set was not shared with or passed on to other organizations, and it was kept confidential (Ethical approval number, authLetter_177807). The primary data were collected as per national and international ethical standards. Each woman provided written consent before data collection. Identification, such as a name, was not recorded to maintain confidentiality.

## Results

### Sociodemographic characteristics of participants

In both surveys, the majority of mothers (47% in 2000 and 49.8% in 2019) were between the ages of 25 and 34 years. In 2000 and 2019, approximately 90% and 70% of children were rural residents, respectively. In 2000, 6% of children were born to mothers who had completed secondary or higher education, compared to 14% in 2019. About 5% of children in 2000 and 54% of children in 2019 were born at health facilities. About 20% of households in 2000 and 38% of households in 2019 had radio or television during interview times (Table 1).

**Table 1:** Sociodemographic characteristics of the participants in Ethiopia based on 2000 and 2019 EDHS data, Ethiopia.

Variables		Frequency N (%)	
		2000 EDHS	2019 EDHS
Region			
	Developed	1970 (94.9)	872 (87.6)
	Developing	67 (3.2)	84 (8.4)
	City Administrative	39 (1.9)	40 (4.0)
Age of mother			
	15–24	638 (30.7)	307 (30.9)
	25–34	977 (47.0)	496 (49.8)
	35–49	461 (22.2)	193 (19.4)
Residence			
	Urban	215 (10.4)	299 (30.0)
	Rural	1861 (89.6)	697 (70.0)
Educational status of the mother			
	No education	1650 (79.5)	450 (45.2)
	Primary	311 (15.0)	406 (40.7)
	Secondary or above	115 (5.5)	140 (14.1)
Religion of mother			
	Christian	1354 (65.2)	438 (64.1)
	Other	722 (34.8)	358 (35.9)
A household has a Radio/TV			
	No	1667 (80.3)	615 (61.7)
	Yes	409 (19.7)	381 (38.3)
ANC Visits during index baby			
	No	1522 (73.3)	251 (25.2)
	1–3 visits	336 (16.2)	306 (30.7)
	4 or above visits	218 (10.5)	439 (44.1)
Place of delivery during index baby			
	Home	1980 (95.4)	461 (46.3)
	Health institution	96 (4.6)	535 (53.7)
PNC visit during index baby			
	No	1960 (94.4)	626 (62.8)
	Yes	116 (5.6)	370 (37.2)
Birth order of index baby			
	1	335 (16.2)	230 (23.1)
	2–3	696 (33.5)	372 (37.3)
	4 or above	1045 (50.3)	394 (39.6)
Sex of baby			
	Male	1066 (51.4)	488 (49.0)
	Female	1010 (48.6)	508 (51.0)
Number of under 5 children in the household			
	≤1	702 (33.8)	430 (43.2)
	>2	1374 (66.2)	566 (56.8)
History of child death			
	No	1838 (88.5)	969 (97.3)
	Yes	238 (11.5)	27 (2.7)

### Full immunization coverage

About 14.62% and 44.18% of children aged 12 to 23 months were fully immunized in 2000 and 2019, respectively. DPT1 coverage increased by 32.36%, but polio1 coverage declined by 4.14% in 2019 as compared to the 2000 EDHS. The coverage of DPT3 and polio3 increased by 40.58 and 26.29%, respectively, in 2019. The drop rate for DPT (DPT3-DPT1) was 22.73% in 2000 and 14.51% in 2019. Surprisingly, the rate of children who did not receive any vaccine increased by 3.14% in 2019 (Table 2).

**Table 2:** Immunization coverage among children aged 12–23 months based on 2000 and 2019 EDHS data, Ethiopia.

Vaccines	In 2000 EDHS	In 2019 EDHS	Point difference (2019–2000)
	Yes (%)	Yes (%)	
Received one dose of DPT (DPT1)	43.97	76.33	32.36
Received three doses of DPT (DPT3)	21.24	61.82	40.58
Received one dose of polio (polio1)	82.5	78.36	-4.14
Received three doses of Polio (polio3)	35.03	61.32	26.29
Received one dose of BCG	45.57	72.83	27.26
Received one dose of Measle	26.96	58.54	31.58
Fully immunized	14.62	44.18	29.56
Not received any vaccines	16.51	19.65	3.14

### Full immunization coverage by participant characteristics

In 2000, 12.3% of children born to women between the ages of 15 and 24 were fully immunized, compared to 39.5% in 2019. One in ten (11.3%) rural children in 2000 and one in three (37.6%) in 2019 were fully immunized. In 2020, 10.4% of children born to mothers with no formal education were fully immunized, compared to 34.7% of children in the same group in 2019. In 2000, nearly one-fourth (23.2%) of children whose mothers had at least one PNC visit were fully vaccinated, compared to two-thirds (66.3%) in 2019.

The chi-square test result showed that in the 2000 survey, full immunization coverage was associated with the mother’s age, residence, mother’s educational status, ANC visits, delivery place, PNC visit, and child death history at a p-value< 0.05. Similarly, in the 2019 survey, the variables residence, mother’s educational status, having radio or television, ANC visits, delivery place, PNC visits, child death history, and the number of under-five children in the household were significantly associated with full immunization coverage (Table 3).

**Table 3:** Distribution of full immunization coverage by explanatory variables among children aged 12–23 months based on 2000 and 2019 EDHS data, Ethiopia.

Variables		Full immunization coverage (%)		Point difference (2019–2000)
		2000	2019	
Region		***	***	
	Developed	13.6	44.3	30.7
	Developing	14.9	26.0	11.4
	City Administrative	67.2	79.5	12.3
Age of mothers’		**		
	15–24	12.3	39.5	27.2
	15–24	12.3	39.5	27.2
	25–34	17.8	43.3	25.5
	35–49	11.1	53.8	42.7
Residence		***	*	
	Urban	43.7	59.5	15.8
	Rural	11.3	37.6	26.3
Educational status of the mother		***	****	
	No education	10.4	34.7	24.3
	Primary	25.6	47.2	21.6
	Secondary or above	45.6	66	20.4
Religion of mother				
	Christian	15.9	45.8	29.9
	Other	12.1	41.3	29.2
A household has a Radio/TV		***	**	
	No	11.2	37.8	26.6
	Yes	28.6	54.4	25.8
ANC Visits during index baby		***	***	
	No	8.7	21.1	12.4
	1–3 visits	25.2	38.5	13.3
	4 or above visits	39.7	61.4	21.7
Place of delivery during index baby		***	***	
	Home	12.3	27.5	14.6
	Health institution	62.7	58.6	-3.3
PNC visit during index baby		*	***	
	No	14.1	31.1	17
	Yes	23.2	66.3	43.1
Birth order of index baby				
	1	18.3	48.4	30.1
	2–3	15.9	46.8	30.9
	4 or above	12.6	39.3	26.7
Sex of baby				
	Male	15.2	43.6	28.4
	Female	14.1	44.8	30.7
Number of under 5 children in the household			*	
	≤1	16.4	51.4	35
	>2	13.7	38.7	25
History of child death		*	***	
	No	15.5	45.3	29.8
	Yes	8.3	3.9	-4.4

Key: * = X^2^ p- value <0.05

** = X^2^ p- value <0.01, and

*** = X^2^ p- value <0.001.

## Decomposition analysis

Full immunization coverage increased by 29.56% (95% CI: 24.84, 34.28) in 2019. It rose from 14.62% (95% CI: 12.43, 17.11) in 2000 to 44.18% (95% CI: 37.19, 51.41) in 2019. The decomposition analysis showed that 74.95% of the explained change was attributable to differences in the composition of explanatory variables between the two surveys. Changes in the composition of women’s age, antenatal and postnatal care visits, the number of under-five children in the household, and child death history between the two surveys significantly contributed to the change in full immunization coverage.

The difference in the composition of four or more ANC visits between the two surveys accounted for 17.06% of the percentage change in full immunization coverage in 2019 (β = 0.0504, 95%CI: 0.0124, 0.0885). Similarly, PNC visits were responsible for 16.90% of the percentage of explained change (β = 0.0450, 95%CI: 0.0202, 0.0797). Furthermore, the difference in the composition of having two or more under-five children in the household (β = 0.0074, 95%CI: 0.0001, 0.0147) and child death history (β = 0.0526, 95%CI: 0.0349, 0.0703) contributed for 2.50 and 17.80% of the percentage of explained change, respectively. On the other hand, the difference in the composition of elderly women (35 and older) between the two surveys has a significant counteracted effect (-2%) on the change in immunization coverage (β = -0.0062, 95% CI: -0.00930, -0.0032). The remaining overall change, 25.05% was attributed to differences in the effects of explanatory variables (coefficient) between the two surveys. The change in the coefficient of child death history was significantly attributed to the counteracting effect (-20.40%) in full immunization coverage over time (β = -0.0603, 95%CI: -0.1160, -0.0047) (Table 4).

**Table 4:** Contribution of the difference in composition and the effect of explanatory variables on the change in full immunization coverage among children aged 12–23 months in Ethiopia between 2000 and 2019 EDHS.

Variables		Change due to the difference in composition of variables (E)		Change due to the difference in the effect of variables (C)		Total (%)
		coefficient	%	coefficient	%	
Region						
	Developed	1		1		
	Developing	-0.0047 (-0.0099, 0.0004)	1.6	-0.0029 (-0.0074, 0.0016)	0.97	2.57
	City	0.0029 (-0.0001, 0.0058)	0.98	0.0005 (-0.0029, 0.0038)	0.15	1.13
Age of mother						
	15–24	1		1		
	25–34	0.0019 (-0.0006, 0.0045)	0.65	-0.0039 (-0.0595, 0.0517)	-1.31	-0.66
	35–49	-0.0062 (-0.0093, -0.0032) ***	-2.11	0.0388 (-0.0017, 0.0792)	13.12	11.01
Residence						
	Urban	1		1		
	Rural	0.0136 (-0.0071, 0.0343)	4.60	0.0508 (-0.0876, 0.1892)	17.18	21.78
Educational status of the mother						
	No education	1		1		
	Primary	0.0200 (-0.0036, 0.0436)	6.77	-0.0043 (-0.0225, 0.0138)	-1.47	5.30
	Secondary+	0.0056 (-0.0057, 0.0168)	1.88	0.0002 (0.0100, 0.0103)	0.60	2.48
Religion of mother						
	Christian	1		1		
	Other	0.0003 (-0.0006, 0.0014)	0.12	0.0216 (-0.0183, 0.0616)	7.32	7.45
A household has a Radio/TV						
	No	1		1		
	Yes	-0.0088 (-0.0250, 0.0074)	-2.99	-0.0123 (-0.0373, 0.0127)	-4.16	-7.15
ANC Visits						
	No visits	1		1		
	1–3 visits	0.0066 (-0.0106, 0.0238)	2.23	-0.0203 (-0.0422, 0.0016)	-6.86	-4.63
	4 or above	0.0504 (0.0124, 0.0885) **	17.06	-0.0038 (-0.0189, 0.0114)	-1.27	15.79
Delivery place						
	Home	1		1		
	Health institution	0.0302 (-0.0194, 0.0798)	10.21	-0.0059 (-0.0138, 0.0019)	-2.01	8.10
PNC visit						
	No	1		1		
	Yes	0.0450 (0.0202, 0.0797) ***	16.90	0.0058 (-0.0032, 0.0148)	1.95	18.85
Sex of baby						
	Male	1		1		
	Female	-0.0002 (-0.0021, 0.0017)	-0.06	0.0074 (-0.0401, 0.0549)	2.50	2.44
Number of under 5 children in HH						
	1	1		1		
	2 or more	0.0074 (0.0001, 0.0147) *	2.50	-0.0361 (-0.1066, 0.0344)	-12.21	-9.71
History of child death						
	No	1		1		
	Yes	0.0526 (0.0349, 0.0703) ***	17.80	-0.0603 (-0.1160, -0.0047) *	-20.40	-2.60
Total		0.2215 (0.1715, 0.2715) ***	74.95	0.0741 (0.0135, 0.1346) *	25.05	100

Key: 1 reference

* significant at <0.05

** significant at <0.01, and

*** significant at <0.001.

## Discussion

Over the last two decades, Ethiopia has achieved great progress in improving child health. However, must cut under-five mortality by three times, starting in 2016, to meet SDG 3 by 2030. Providing vaccines to all children in need is one of the key strategies to prevent vaccine-preventable diseases, which account for the lion’s share of child deaths in Ethiopia. However, full immunization coverage remained low and progress is slow. The objective of this study was to assess the change in childhood full immunization coverage in Ethiopia and identify factors attributed to change using two EDHS data conducted in 2000 and 2019. It is very important to achieve access to vaccination services for everyone everywhere agenda and end vaccine-preventable child mortality [16].

The finding of this study revealed there was a statistically significant change in immunization coverage between the two surveys, it has increased from 15% in 2000 to 44% in 2019. Previous studies also documented that full immunization coverage increased over time [20–24]. The authors agreed that the rising immunization coverage over time in Ethiopia may be attributed to increasing enabling factors such as enhancing women’s education status, service accessibility, level of awareness about immunization among women, maternal health service utilization, and primary health care coverage recently.

Efforts to reduce adverse outcomes for pregnant women and newborns over the past decades, such as the MDGs and more recently, the SDGs agenda have improved maternal health services uptake in Ethiopia [11]. Maternal health services utilization provides the opportunity for women to get counseling and education on the importance of immunizing their children against vaccine-preventable diseases, allowing them to be more confident in utilizing childhood immunization services [20]. Previous studies documented that childhood full immunization coverage was higher among women who had an ANC visit [25–28], delivered at a health facility [25, 28], and had a PNC visit [26, 28].

Improving women’s education could be another major factor in increasing complete immunization coverage in Ethiopia; the percentage of educated women nearly tripled between the two surveys [11, 12]. Education plays an important role in increasing female autonomy, and access to information through various media enables them to be aware of the benefits of immunization. Previous studies have found that a mother’s education status was a strong predictor of complete immunization [21, 25–28]. Besides, the media has a positive impact on a child’s immunization status. In recent years, governmental and non-governmental organizations have expanded their maternal and child health-related programs on television and radio, presumably enhancing mothers’ understanding of immunization.

Far distances to nearby health facilities, lack of transportation, poor quality immunization information, and frequent vaccine shortages are the main barriers to the EPI program in Africa [9]. Ethiopia is striving to address these concerns by launching the Health Extension Program (HEP), in 2005 [14], which has played a significant role in improving maternal and child health by offering services at the community level. HEP has a substantial impact on achieving primary healthcare coverage in Ethiopia, which enhances the accessibility and availability of the service, particularly in rural areas. Health Extension Workers (HEWs) who work under HEP give health awareness and services on child health like immunization and management of common childhood illnesses, as well as maternal health care, at the kebele (the lowest level of administration) and the household level. Evidence showed that HEP increases maternal and child health service uptake by raising awareness and providing services at the community level [29–31]. Previous studies also have shown that mothers’ awareness and knowledge of childhood immunization are significant predictors of the completion of childhood immunization [32–34].

In this study, the decomposition analysis showed the change in full immunization coverage between the two surveys was significantly attributed to both a difference in the composition and the effect of explanatory variables. About three-fourths (75%) of the explained change was attributed to the difference in the composition of explanatory variables. The difference in composition of the age of the mother, ANC visits, PNC visits, number of under-five children, and child death history significantly contributed to the change in full immunization coverage over time.

The change in the composition of elderly women (35 and older) between the two surveys had a substantial counteracted effect (-2%) on the change in full immunization coverage. The reason could be related to the fact that older women might have less knowledge about modern health services and the value of modern medicine since the improvement in educational opportunities available to women in recent years [35]. Another possible justification may be the high birth order among older women, which has a negative effect on healthcare utilization. A study showed that mothers with higher parity were less likely to immunize their children [36]. On the contrary, studies showed that young mothers were less likely to complete their child’s immunization [36, 37].

In this study, about 3% of the percentage change in full immunization coverage was due to the change in the composition of having two or more children in the household. Having two or more under-five children in the household should be a challenge to women because all under-five children need care and help from their mothers, and as a result, they may not have time to take their children to the immunization center.

This study revealed that about 18% of the percentage change in full immunization coverage was due to a change in the composition of women with a child death history over time. This may be due to people don’t recognize a link between not being immunized and an increased risk of childhood death. A previous study also showed that maternal health services were underutilized among mothers who had lost a preceding child [38].

The difference in the composition of four or more ANC visits between the two surveys accounted for 17% of the percentage change in full immunization coverage from 2000 to 2019 compared to not having ANC visits. This finding was supported by previous studies [23, 24]. The possible justification might be that frequent antenatal care services may be a crucial signal to show women that they are accessible to healthcare facilities and that they can build a good rapport with healthcare providers. Such interactions allow healthcare providers to encourage and educate women to prioritize healthcare for themselves and their children [39].

Similarly, the difference in composition of having a PNC visit between 2000 and 2019 accounted for 17% of the percentage explained change in full immunization coverage. The reason for this may be that PNC services provide an avenue to receive information on childhood immunization and initiate vaccines, which could later enhance compliance with the immunization program [34, 40].

The difference in the effect of the explanatory variables (coefficient) between the two surveys accounted for the remaining 25% of the change in full immunization coverage. The difference coefficient among women having a history of child death across the two surveys significantly reduced the change in full immunization coverage by 20%. Previous children’s deaths could have pushed mothers to immunize their children if they believed the death was due to not being immunized or discouraged them if they believed the death was due to vaccination. This indicates the need for further study to explore the role of experiencing child death on childhood immunization.

The data for this study comes from a large, nationally representative survey. This finding revealed a change in full immunization coverage over two decades and identified the factors contributing to the change. This will help increase immunization coverage in Ethiopia by focusing on contributory factors. Some important vaccination-related variables, however, missing from this analysis because they were not collected at the primary source. Infrastructure and human resources are major factors that contributed to low immunization coverage [9] but were not addressed in this study.

## Conclusion

The finding of this study revealed that there was a statistically significant change in full immunization coverage in the time between 2000 and 2019, but the progress is slow, increasing by only 1.5% every year in the last two decades. The change was attributed to both differences in composition and effect of explanatory variables, but the majority, which was about 75% of the percentage change contributed to the difference in composition of the explanatory variables. The difference in composition of the age of the mother, ANC visits, PNC visits, number of under-five children in the household, and mother with a child death history were significantly contributed to the change in immunization coverage over time. The increasing composition of women having four or more ANC and PNC visits enhances full immunization coverage in Ethiopia, emphasizing the need to expand ANC and PNC service availability and accessibility to all women in need of them. This study also emphasis the need to improve access to and availability of family planning services, as well as provide information on high maternal risky fertility behaviors. The rest 25% of the observed change in immunization coverage between the two surveys was attributed to the difference in the coefficient over time. This study demonstrated that change in the coefficient for women with a history of child mortality had a significant counteracting effect on the change in immunization coverage. This suggests a necessity for raising awareness and delivering education about vaccines to them.
